# CNN-LSTM-Based Prognostics of Bidirectional Converters for Electric Vehicles’ Machine

**DOI:** 10.3390/s21217079

**Published:** 2021-10-26

**Authors:** Gabriel Rojas-Dueñas, Jordi-Roger Riba, Manuel Moreno-Eguilaz

**Affiliations:** 1 Electrical Engineering Department, Universitat Politècnica de Catalunya, Rambla Sant Nebridi 22, 08222 Terrassa, Spain; gabriel.esteban.rojas@upc.edu; 2 Electronics Engineering Department, Universitat Politècnica de Catalunya, Rambla Sant Nebridi 22, 08222 Terrassa, Spain; manuel.moreno.eguilaz@upc.edu

**Keywords:** power converters, electric vehicles, fault diagnosis, accelerated aging tests, artificial neural networks

## Abstract

This paper proposes an approach to estimate the state of health of DC-DC converters that feed the electrical system of an electric vehicle. They have an important role in providing a smooth and rectified DC voltage to the electric machine. Thus, it is important to diagnose the actual status and predict the future performance of the converter and specifically of the electrolytic capacitors, in order to avoid malfunctioning and failures, since it is known they have the highest failure rates among power converter components. To this end, accelerated aging tests of the electrolytic capacitors are performed by applying an electrical overstress. The gathered data are used to train a CNN-LSTM model that is capable of predicting the future values of the capacitance and the equivalent series resistance (ESR) of the electrolytic capacitor. This model can be used to estimate the remaining useful life of the device, thus, increasing the reliability of the system and ensuring an adequate operating condition of the electric motor.

## 1. Introduction

Electrical machines are being broadly applied in different fields, including electric automobiles, aviation, trains, ships, or industry among others, playing a key role in these applications. However, faults in electrical machines have damaging effects, posing a risk to the safety of the involved applications [1]. Therefore, the application of condition monitoring strategies is a must. It is based on applying different methods to identify changes in a system because of fault development or the degradation of the state of health (SoH), thus generating an alarm to indicate the occurrence of a failure [2]. To apply effective on-line condition monitoring approaches, it is necessary to develop methods for detecting degraded or anomalous operational modes in the early stage, much before the development of important faults, while minimizing false alarm events [3,4].

Therefore, predictive maintenance of electrical machines and drives is gaining popularity in different fields, from electric traction to industrial activities. There are several reasons, from the high capital cost and central importance electrical machines play in the process, to the heavy economic losses that unexpected faults can generate because of needed machine or parts replacements, loss of production due to its unavailability, or property damage to property and people injuries [5]. The imperious requirement of manufacturing more competitive equipment, together with the progress of digital technologies, has facilitated the acquisition of operational data, which can be processed by means of machine learning (ML) methods to extract valuable information to apply data-driven diagnosis and maintenance approaches [6].

Power converters often operate in closed-loop systems, and thus, any change in one of their components will modify the operating point of other constituents and the resultant thermal stress, thus affecting overall system reliability. This change tends to accelerate the aging process of the affected and the remaining components of the converter. It is generally accepted that electronic components exhibit a bathtub failure curve. It includes three stages, i.e., an initial infant mortality stage followed by a constant failure rate region and ending in the wear-out period. However, several deviations with respect to the bathtub curve have been reported [7]. It is known that capacitors and power switches are the power converter components with the highest failure rate [8,9], capacitors accounting for around 30% failures in power converters [10]. Reliability plays a key role in power electronic systems, especially for those applications in which availability is a critical parameter, so that even under faulty conditions the application should be able to operate [11].

Capacitors are among the most critical components of power converters. They have a huge influence in the final cost, performance, and size of such electronic systems, although they exhibit the highest degradation rates and shorter lifespans among all power converter components [12]. Therefore, condition monitoring plays a key role to estimate the health status of capacitors and to apply predictive maintenance tasks for ensuring stability in the operation of power converter systems. The equivalent series resistance (ESR) and the capacitance of the capacitor are two widely used parameters for evaluating the health status of capacitors. Unlike the ESR, the capacitance of a capacitor is suitable for health monitoring of various types of capacitors; therefore, it is more preferable for large-scale systems [12].

In the last years, artificial neural networks (ANNs) have been widely applied to identify and estimate the values of different parameters of electrical systems, including power converters. In [13] ANNs are applied for a parametric fault detection in DC-DC converters, while in [14] a long short-term memory neural network (LSTM-NN) was applied to estimate the remaining useful life (RUL) of the converter. An accurate prediction of the RUL enables to apply cost-effective maintenance plans by anticipating when the system under observation must be replaced, thus greatly reducing premature failure risk and the associated undesirable effects, while optimizing power system operational efficiency [15,16]. In [17] a LSTM-NN was applied to model the transient behavior of a DC-DC power converter used in mild hybrid electric vehicles. In [18] a recursive artificial neural network, in this case a nonlinear autoregressive exogenous neural network (NARX-NN) was applied for reproducing the behavior of a DC-DC buck converter at the expense of the time required to train the network. Wavelet artificial neural networks (WA-NNs) have been also applied for this purpose. They apply a wavelet transform to the NN input signals for extracting time-frequency features, thus simplifying the data structure and reducing the associated computational requirements [19]. It is important to note that ML methods do not require accurate mathematical models of the system they are emulating and are usually highly adaptable. However, they require sufficient past data to train the algorithm, representing a broad range of operating conditions. NNs also require to set the value of different parameters or hyper-parameters, and jointly with the greater computational cost compared to other methods, it may hinder their implementation in practical engineering applications [11].

Mission-critical power electronics systems, including renewable energy integration, data center power delivery, and motor drives applications, require high reliability and availability of service [1,2]. In many of these scenarios, techniques for fault prognosis are commonly employed, that is, methods for actively monitoring the system condition and predicting when failures will occur. Parameter identification is a central technology enabling fault prognosis, or identifying the values of system parameters in real time and online. By tracking the values of important system parameters in real time, operators can actively monitor the overall health of a system and anticipate when maintenance or repairs will be needed. Moreover, fault prognosis can be achieved by monitoring if estimated parameter values are above or below an accepted tolerance range [20]. Furthermore, an online state of health estimation of electrical devices is useful to estimate the RUL with high accuracy [21]. In this case, the RUL is continuously updated based on the actual state of the device. The auto-regressive integrated moving average (ARIMA) method is widely used to estimate the RUL of several devices, which is a data-driven approach. It has been used to estimate the RUL of aircraft engines [22], lithium-ion batteries [23], and machine health condition, among others. In [24] a method to estimate the RUL of an electrolytic capacitor based on a Kalman filter forecasting algorithm is presented. It proposes an invasive degradation procedure and calculates the end of life of the capacitor based on the aging curve.

This paper presents the design, implementation, and experimental validation of a novel method for fault prognosis for power converters using a deep learning-based parameter identification approach. The parameter identifier uses a generalized gradient descent algorithm to compute real-time estimates of the analyzed parameters (capacitance and ESR) [20]. Since the capacitor is perhaps the most critical element in power converters, the evolution of two parameters, i.e., the ESR and the capacitance, is used to determine and forecast the future condition of the capacitor and thus, of the power converter. To this end, the capacitor was aged by means of accelerated aging tests based on applying an electrical overstress, during which the parameters of the capacitor were monitored. The deep learning method used to forecast the values of the capacitance, and the ESR of the electrolytic capacitor is based on combining a one dimensional convolutional neural network (CNN) and a long short-term memory neural network (LSTM). The proposed prognostics approach combines white-box parameter estimation and a neural network structure. Whereas the parameter estimation stage is used to determine the capacitance and the ESR value of the electrolytic capacitor, the deep learning neural network is applied to forecast the future values of these parameters. The effectiveness of the method is proved from theoretical analysis, simulation, and experimental verification, respectively. This paper contributes in the state-of-the-art in several ways. First, it proposes a non-invasive aging test procedure of the output electrolytic capacitor that is found in DC-DC converters. This test outperforms the degradation procedure presented in [25] since it does not require to stop the test to measure the parameters of the capacitor using a LCR meter. The proposed aging test only uses the signals at the input and output terminals of the converter for a continuous estimation of the capacitor parameters. Second, the CNN-LSTM neural network topology has not yet been applied in this field, outperforming other state-of-the-art approaches found in the technical literature. The proposed model is capable to learn from the complex patterns of the time-series and forecast the future values with high accuracy.

Section 2 describes the powertrain of an electric vehicle, emphasizing on the DC-DC bidirectional converter. It also proposes an accelerated aging test to degrade the output capacitor of the converter. Section 2 also details the prognostics approach proposed in this study. It presents how the capacitor parameters are estimated during the degradation process and how these data are used to generate a CNN-LSTM model capable of predicting the future values of the capacitance and the ESR. Section 3 shows and analyzes the obtained experimental results. Finally, Section 4 presents a discussion based on the main findings of this study.

## 2. Materials and Methods

### 2.1. Powertrain of an Electric Vehicl Jmghj

**Figure 1:**
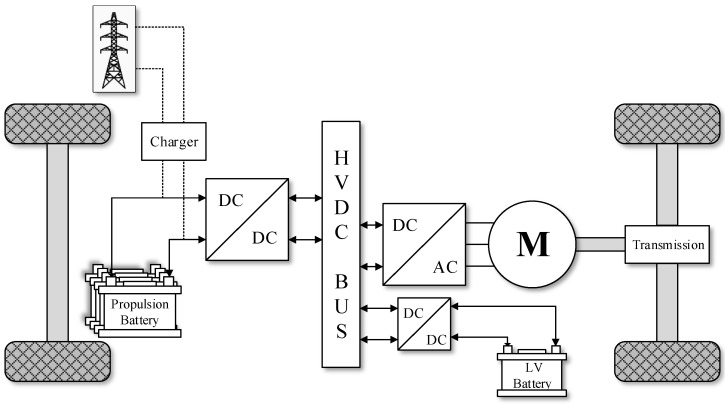
Powertrain of an electric vehicle.

The powertrain of electric vehicles consists of various power converters that are in charge of delivering the power generated by the battery pack to the electric motor and the electronic loads of the vehicle [26]. The architecture of the electrical system may change depending on the specific characteristics of the vehicles. Nevertheless, there are specific components that can be found in most of the electric and hybrid electric vehicles [27]. Figure 1 shows a block diagram of a typical powertrain of an electric vehicle, which includes the different elements involved in the energy conversion process. Typically, there are three types of converters in the electrical system. These are a DC-DC high voltage bidirectional converter, a DC-DC low voltage bidirectional converter, and a three-phase inverter that feeds the electric motor. However, in technologies that also consider a fuel cell as an energy source, another converter is included.

The DC-DC converters used in electric vehicles must meet certain conditions in order to guarantee a proper operation of the vehicle and to offer a reliable and efficient conversion process [26]. Some of these essential requirements include light weight, small volume, high efficiency, low electromagnetic interference, low ripple or control of the current flowing through the converter, among others [26]. Thus, switched mode power converters arise as a solution, since they are capable of fulfilling these requirements with high performance. These type of converters are widely used in multiple applications and provide high reliability to the electrical system of the vehicle.

**Figure 2:**
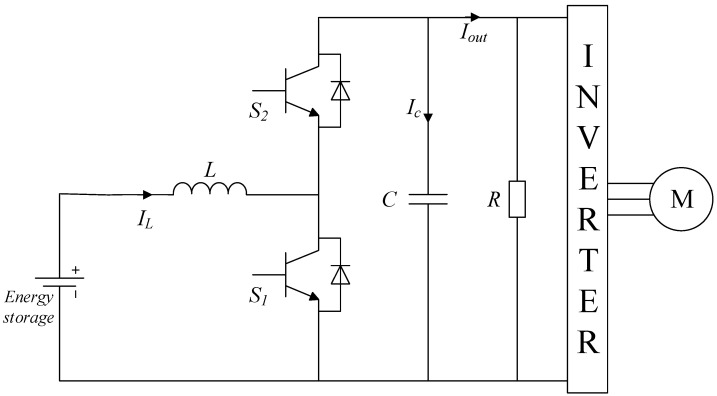
Typical topology of a bidirectional DC-DC converter used in electric vehicles.

Since power converters play a key role in the propulsion of electric vehicles, it is necessary to apply a real time estimation of their health status in order to guarantee a correct operation of the powertrain and prevent possible failures. A brief analysis of the DC-DC converters used in these vehicles is necessary to better understand the possible failures that they may incur. The most critical power electronics related element of the powertrain shown in Figure 1 is the bidirectional power converter, because it feeds the electrical machine that propels the automobile. Figure 2 presents the typical architecture of a DC-DC bidirectional converter used in automotive applications [28]. It is seen that the converter links the energy storage system to the electric motor of the vehicle, while allowing the current flowing in both directions. Among the elements of the converter shown in Figure 2, the transistors and the electrolytic capacitor are the most likely to fail [25], being the capacitor the main reason of power system breakdowns [25]. The degradation of these capacitors results in an undesired voltage ripple that affects the efficiency of the power converter. The continuous operation of a degraded capacitor may lead to an irreversible damage of the DC-DC converter. Thus, this study focuses on the prognostics of the faultiest element of the bidirectional converter, which is the electrolytic capacitor.

#### Electrolytic Capacitor

Capacitors are well known for being a component that protects the drives and distribution systems from the perturbations generated by heavy dynamic loads like the motor of an electric vehicle. However, electrolytic capacitors are also recognized for being the originators of failures in important power supply systems, such as actuators, avionics equipment, or power electronics devices, among others [29]. Therefore, the prognostics and state-of-health estimation of these elements becomes imperative in power systems that rely on their proper operation.

Normally, the electrolytic capacitor is modeled with four passive elements, which are the capacitance, equivalent series resistance (ESR), equivalent series inductance (ESL) and the leakage resistance. However, this study only considers the capacitance *C* and ESR in the state-of-health estimation of the electrolytic capacitor, since these parameters are the ones with a greater impact on the capacitor performance [29].

**Figure 3:**
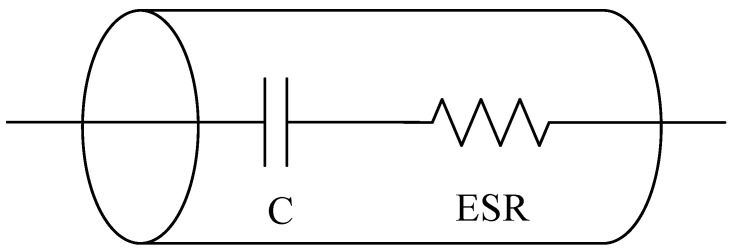
Electrical model of an electrolytic capacitor.

Figure 3 presents the simplified electrical model of an electrolytic capacitor. The degradation of this type of capacitors results in a decrease of the capacitance and an increase of the ESR, which eventually affects the performance of the electrolytic capacitor. According to the standard MIL C 62 F, the capacitor is considered as faulty when the value of the capacitance drops 20% below the value obtained in the manufacturing process, and/or when the ESR increases is above 280% of its initial value.

The main factors that contribute in the degradation process of electrolytic capacitors are the temperature, vibration, pressure, overvoltage, humidity, pressure, and a high current ripple [30]. This study aims to analyze the overvoltage effect on the degradation of the capacitor. Charging the electrolytic capacitor with a voltage above its rated value leads to the evaporation of the electrolyte, an increase of the internal pressure as well as of the leakage current [29]. The electrical overstress of the capacitor results in an increase of its internal temperature because the current flow rises drastically, and consequently, the electrolyte evaporates and the ESR increases. In addition, the internal pressure of the capacitor increases due to the chemical reactions that occur when it is subjected to charging cycles at a high voltage. The following Subsection presents the accelerated aging tests carried out to degrade the electrolytic capacitor.

### 2.2. Accelerated Aging Tests

This study aims to explore the effects of high voltage in the electrolytic capacitors used in DC-DC converters. When the operating voltage of the converter is above the rated voltage of the capacitor, the capacitor tends to degrade, and it may lead to irreversible damage that alters the converter behavior. Thus, an aging test is proposed in order to charge and discharge the capacitor in recurrent cycles. To this end, it is necessary to apply a periodic square signal for several hours, with an amplitude higher than the nominal voltage of the capacitor. The expected outcome of this aging test is an increase of the ESR and a decrease of the capacitance. Although the aging test of the electrolytic capacitor does not involve the converter, once the degradation process has been completed, a comparison contrasting the DC-DC converter performance with the initial values of the capacitor and with the degraded capacitor is done.

**Figure 4:**
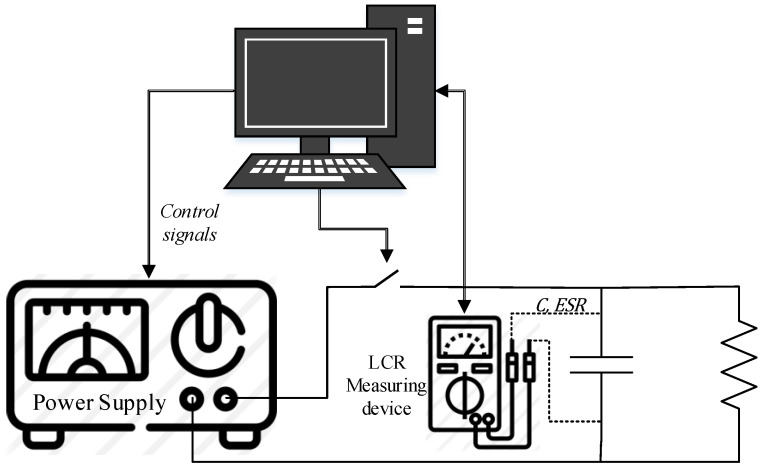
Accelerated aging test data acquisition system.

The accelerated aging test proposed in this paper is based on the experiment detailed in [29,31]. A DC power supply feeds the electrolytic capacitor by applying a voltage that is 20% higher than the nominal value specified by the manufacturer, until the capacitor is fully charged. Then, the power supply switches off and the capacitor is discharged through a resistor connected to it. This cycle repeats for several times until the capacitance is below a predetermined value. Meanwhile, the values of the capacitance and the ESR are measured constantly in order to have a rich dataset. Figure 4 presents the accelerated aging experimental setup. The degradation emulates the long term behavior of an electrolytic capacitor in a DC-DC converter.

Figure 4 shows that the experiment is controlled by a computer, which is in charge of sending orders to the equipment involved in the data acquisition process. Concretely, it controls the voltage delivered by the power supply, the RLC measuring equipment, and a switch that connects or disconnects the capacitor from the DC power supply. The data is acquired every 30 min. In order to have an accurate measurement of the electrolytic capacitor parameters, it is necessary to disconnect the power supply.

#### Laboratory Setup

**Figure 5:**
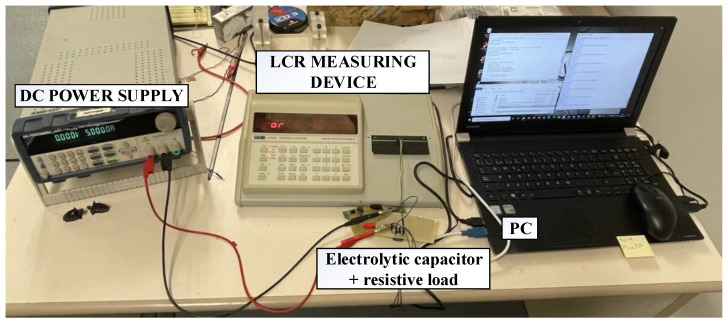
Experimental setup of the accelerated aging test.

The data acquisition system shown in Figure 4 was implemented in the laboratory. The electrolytic capacitor used in the degradation process has a nominal capacitance of 220 µF, an ESR of 300 mΩ, a rated voltage of 7.56 V, and it is manufactured by Panasonic. This capacitor can be found at the output of different DC-DC converters such as the TPS40200EVM-002 step-down converter manufactured by Texas Instruments. In order to discharge the capacitor, a 100 Ω resistor was connected in parallel to the electrolytic capacitor. Figure 5 shows the experimental setup implemented in the laboratory.

**Figure 6:**
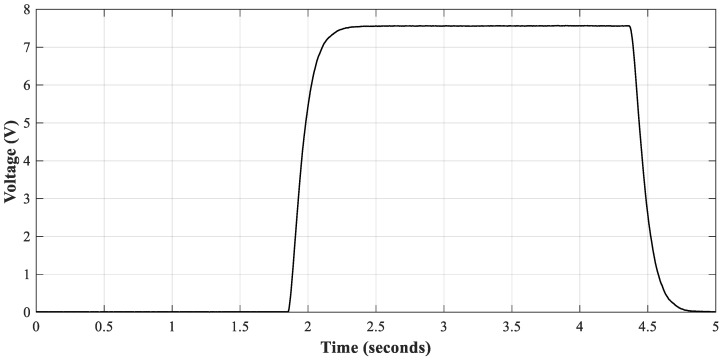
Charging and discharging cycle of electrolytic capacitor.

The DC power supply used in this process is the BK Precision 9205 (BK Precision Corporation, Yorba Linda, CA, USA), while the measuring device is the LCR400 Precision LCR Bridge, manufactured by Thurlby Thandar Instruments. The voltage applied to the capacitor is 7.56 V, which is 1.2 times higher than its nominal voltage. The switching frequency of the power supply is 0.2 Hz, which implies that the capacitor is charged during 2.5 s and discharged during the same amount of time. Figure 6 shows the current and voltage waveforms during the charging and discharging processes.

### 2.3. Proposed Approach

**Figure 7:**
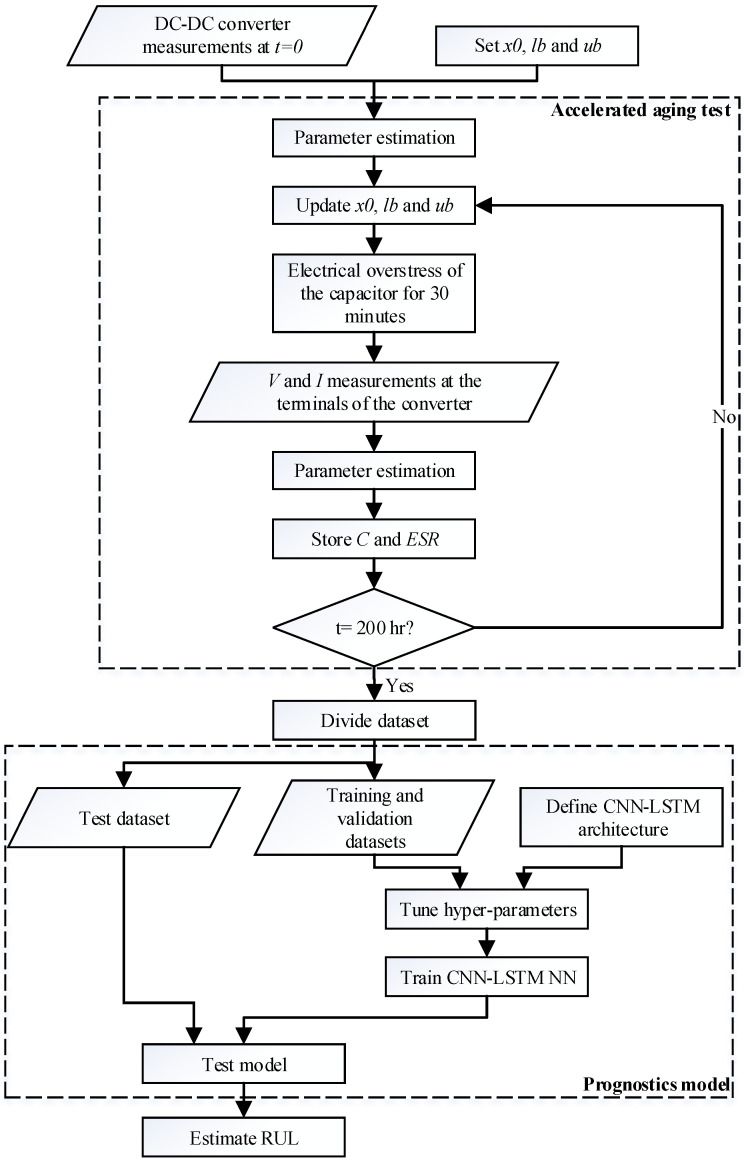
Proposed approach.

The proposed prognostics method combines a white-box parameter estimation method and a neural network structure. The first one is used to determine the capacitance and ESR value of the electrolytic capacitor, while the second one aims to forecast the future values of these parameters. The importance of this approach is that during the whole process, it just requires the signals measured at the input and output terminals of the DC-DC power converter. Figure 7 presents the proposed procedure.

As depicted in Figure 7, the first step is to measure the signals and to estimate the parameters of the power converter when it operates under healthy condition. These parameters are estimated based on an optimization method, where the variables are initialized with an initial value (*x*_0_) and the maximum (*lb*) and minimum (*ub*) values are set. Then, the degradation process of the electrolytic capacitor starts, and every 30 min the parameters are identified based on the non-invasive measurements of the converter. This is done until the accelerated aging test reaches a certain limit time. At the end of each iteration, the capacitance and ESR values are stored, while the initial, minimum and maximum values of the parameters are updated for the next identification. As mentioned in Section 2.2, the values of the capacitance and resistance are also measured using a specialized device to validate the accuracy of the parameter estimation method.

Once the degradation test has finished, the next step is to obtain a model that is able to predict the future values of the capacitor parameters. To this end, the stored data is split into three different datasets, i.e., the training, validation and test sets. The first two are used to train the CNN-LSTM neural network, whereas the test data set is used to determine the accuracy of the predictions made by the model. The architecture of this neural network has to be defined and the hyper-parameters and simulation parameters must be selected before training the model. Finally, the RUL of the capacitor is estimated using the trained model. As mentioned in Section 2, the electrolytic capacitor is considered damaged when the capacitance decreases to the 80% of its initial value or when the ESR reaches the 280% of its nominal value. The CNN-LSTM model is used to estimate the time required to reach the aforementioned values, thus anticipating the time to failure.

The following Subsections present a detailed explanation of the optimization and machine learning methods used in this study.

#### 2.3.1. Parameter Estimation Based on Nonlinear Least Squares Optimization

The optimization algorithm used to estimate the parameters of the DC-DC converter is based on the approaches proposed in [32,33]. It uses the trust-region reflective nonlinear least squares algorithm (TRRNLS) to find out a set of parameter values that minimize an objective function, which in this case is the fitting error. Given that the algorithm deals with nonlinearities, it is especially useful to estimate the parameters of a switched mode DC-DC converter. The variables to be optimized are defined as the vector *x*, which includes the parameters of the converter. The objective function of the optimization problem is obtained by comparing the measured and estimated voltage and current signals of the converter, and it is defined as follows:

(1) $$\underset{x}{\min}E\left(x\right)$$

(2) $$E(x)=\sum_{i=1}^{n}e_{i}^{2}\left(x\right)=\sum_{i=1}^{n}\left({\left(V_{in}^{est}\left(t\right)-V_{in}^{meas}\left(t\right)\right)}^{2}+{\left(V_{out}^{est}\left(t\right)-V_{out}^{meas}\left(t\right)\right)}^{2}+{\left(I_{in}^{est}\left(t\right)-I_{in}^{meas}\left(t\right)\right)}^{2}+{\left(I_{out}^{est}\left(t\right)-I_{out}^{meas}\left(t\right)\right)}^{2}\right),t=iT$$

*E*(*x*) is the error function that is minimized during the process, *V* the voltage, and *I* the current at the terminals of the DC-DC converter. The problem is constrained by the minimum and maximum values of the parameters to be optimized, which can be expressed as lb<x<ub. The TRRNLS optimization generates a trust region that is reduced after each iteration in order to find a set of parameters that reach a local minimum. This trust region is generated after the initial point $x_{0}$ is defined. The algorithm is in charge to find the next point, within a certain neighborhood *N*. The new point must produces an error lower than the previous one, otherwise it does not change, and the trust region is reduced. Thus, the iteration step $s_{k}=x_{k}-x_{k-1}$ is calculated at every iteration. Its length depends on the space given by the minimum and maximum values of the variables to be identified. The process of searching the points in the boundaries of the neighborhood *N* is done by applying the reflective line search. The space int(N) is restricted to two dimensions in order to enhance the speed of the algorithm, since the computation of eigenvalues, eigenvectors, Jacobian and Hessian matrices is simpler when the dimensionality is reduced. It is important to specify a proper initial point as well as the parameters boundaries because they determine the trust-region and step length.

As it is proposed in [32], the signals used to tune the converter parameters are the input and output voltages of the converter under steady-state, and when a load change occurs. The steady-state signals are used to find the parameters that affect the ripple of the signals, while the transient signals are required to estimate the values that have an influence in the transient response of the converter. In the method proposed in Figure 7, the parameters of the DC-DC converter are estimated before the accelerated aging test starts. For this case, the initial point and the lower and upper boundaries are selected based on a-priori knowledge. Thus, the seed point is chosen depending on the parameter (the inductor value is set to 1 µH) and the minimum and maximum values are chosen to cover four orders of magnitude (0.01 µH < L < 100 µH). After the degradation process starts, the parameters are estimated every 30 min and the initial point of the estimation is the one obtained in the previous iteration, while the boundaries are set to $0.8x_{t-1}<x_{t}<1.2x_{t-1}$. This means that the parameter estimation process during the accelerated aging test lasts way less than the initial identification of parameters because the solution space is smaller.

#### 2.3.2. Capacitance and ESR Forecasting Based on CNN-LSTM

The deep learning method used to forecast the values of the capacitance and the ESR of the electrolytic capacitor is based on combining a one dimensional convolutional neural network (CNN) and a long short-term memory neural network (LSTM). Recurrent neural networks such as LSTM are widely used in the analysis of time-series because they are able to learn the sequential dependencies of the data and use them to predict future values [34]. On the other hand, CNNs are widely used in image classification problems since they are capable of obtaining complex features of data that contain significant information [35]. However, over the last years, the CNNs are being used in time-series related problems since they allow finding relationships between the different time dependencies of the data with a good accuracy. The modeling technique used in this study integrates a one dimensional CNN with a LSTM-NN in order to predict the future values of the capacitance and ESR of the electrolytic capacitor. The main advantage of combining convolutional and LSTM layers is that the model learns the long term dependencies of the complex features of the time-series, which allows the neural network to replicate with high accuracy the behavior of the training data [36].

**Figure 8:**
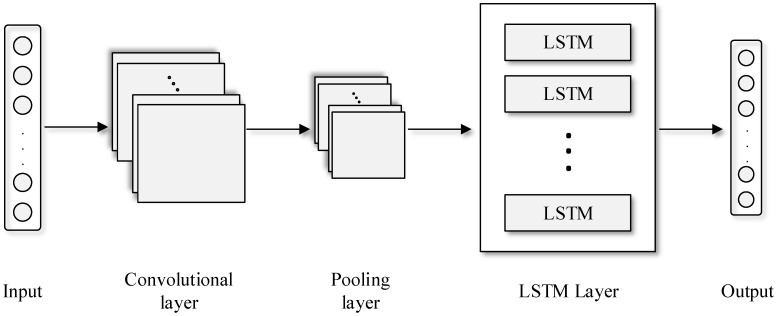
CNN-LSTM NN architecture.

Consequently, the proposed model aims to solve the regression problem of forecasting the future values of the capacitor parameters. A neural network consisting of a combination of a one dimensional CNN and a LSTM is used. Figure 8 shows the neural network architecture proposed in this work. It is seen that the input data inputs the convolutional layer, where the complex features are extracted. Then, the resulting maps input the pooling layer and the obtained output is the input of the LSTM layer, which generates the variable values for the next *m* time steps.

First, the inputs and outputs of the neural network must be defined. This study proposes as input the *n* previous steps of the variable to be predicted (*C* or ESR), while the output considers the *m* future steps of this variable. For instance, if *n* is set to 15 and *m* to 5, the CNN-LSTM NN estimates the next 5 values of the variable *x* based on the last 15 values. The number of samples used to train the model is given by the total length *l* of the dataset and the selected *n* and *m* values, which can be calculated as follows,

samples=l−(m+n)+1

where the timespan of the first, second and third samples are, respectively, $\left(t_{1},t_{m+n}\right)$, $\left(t_{2},t_{m+n+1}\right)$ and $\left(t_{l-m-n},t_{l}\right)$.

Regarding the structure of the neural network, the convolutional layer applies filters or kernels to the input data to obtain a feature map that contains the most significant information of the time-series [35]. The size and number of kernels define the complexity of the feature map. Equation (3) explains how the output of this layer is calculated,

(3) $$C_{k}=W_{k}*x$$

where $C_{k}$ is obtained from the convolution between the input data x and the feature map $W_{k}$, while k refers to the feature map number [35].

**Figure 9:**
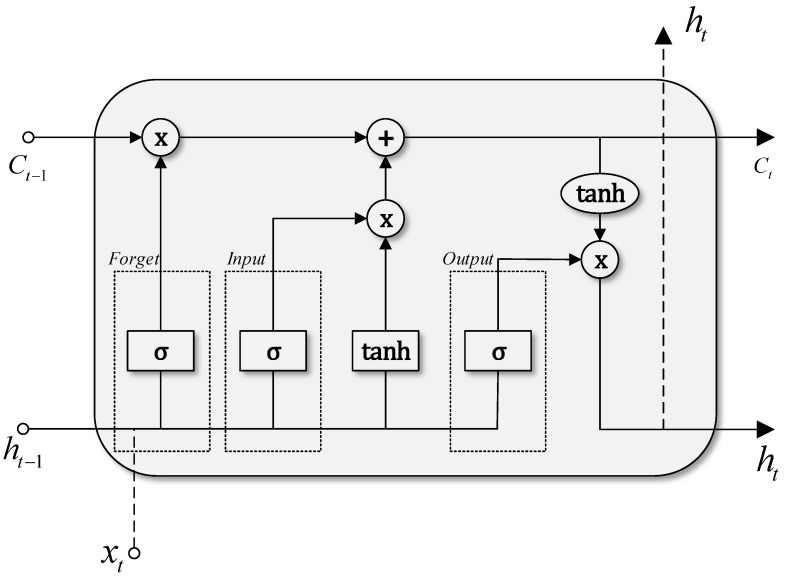
LSTM memory cell.

Usually, the spatial dimension of the feature map imposes a high computational burden during the training process. Therefore, a pooling layer is required in order to reduce the map dimensionality by discarding irrelevant data and keeping the important information [35]. This is done by separating the feature map in different sets, and propagating the maximum (Max pooling) or the average value (Average pooling). Once the pooling layer has extracted the most relevant information of the feature maps, the data enters the LSTM layer. This layer contains memory cells where multiple operations are carried out to generate an output value. The number of cells is the same as the number of time steps of the data analyzed. Each cell contains three gates (input, output and forget) that generate an output by removing irrelevant information and keep the data that is important to minimize the prediction error [37]. Figure 9 shows the architecture of the LSTM memory cell, where the three gates are differentiated. It also shows that multiple pointwise operations occur inside the cell. Figure 9 also shows that a sigmoid activation function is applied, which outputs normalized values between 0 and 1.

Given the structure of the CNN-LSTM model, the next step is to train the neural network using a set of parameters and hyper-parameters and the training data, which in this case is the evolution of the capacitance and ESR with time. The hyper-parameters that have a greater impact on the performance of the model require to be tuned. These are the kernel size, number of neurons of the LSTM layer, and the values of *n* and *m*, which are selected based on a random search algorithm. A total of 100 neural networks were trained for a fixed number of iterations, and the one with the lowest root mean squared error (RMSE) was chosen. Other parameters such as the learning rate, number of filters, gradient decay factor are also defined. Once the hyper-parameters are optimized, the CNN-LSTM is trained. To this end, a large epoch number is defined and the model is trained, calculating the RMSE at every epoch. An early stopping criteria is set. It finishes the training process when the RMSE increases in a row of 3 epochs. After the model is trained, it is tested with a new dataset and the overall accuracy is obtained.

## 3. Results

This section presents the implementation of the approach proposed in Figure 7 to a real DC-DC converter, the results of the accelerated aging tests, and the forecasting of the future values of the capacitance *C* and ESR. The proposed prediction method is compared against four approaches that are used to model time-series related problems. These methods are the nonlinear autoregressive exogenous neural network (NARX NN), a single LSTM NN, a one dimensional CNN, ARIMA and a Kalman filter algorithm [25]. The hyper-parameters of the first three models were tuned by applying a random search algorithm. The NARX-NN has one hidden layer with 8 neurons and the number of delays is equal to 3. The LSTM-NN has one hidden layer with 24 neurons, a learning rate of 0.021 and a gradient decay factor of 0.977. For the case of the 1DCNN, the learning rate is equal to 0.018, it has one convolutional layer with 7 filters and a kernel size of 3. The ARIMA model parameters were (*p*, *d*, *q*) equal to (2, 1, 2), while for the Kalman filter approach, the parameters were selected according to [25].

As mentioned in Section 2.2, the DC-DC converter used is the Texas Instruments TPS40200EVM-002. Despite the fact of not being a bidirectional converter, the chosen device has the same working principle and passive elements as the step-down mode of a bidirectional converter. Also, this evaluation module results useful in the degradation process of the capacitor since it is not packaged, which allows the connection and disconnection of the capacitor. The electrolytic capacitor used in the accelerated aging test is the Panasonic EEEFK0J221AP, with nominal values C=220 μH,ESR=360 mΩ with a tolerance of ±20%.

**Figure 10:**
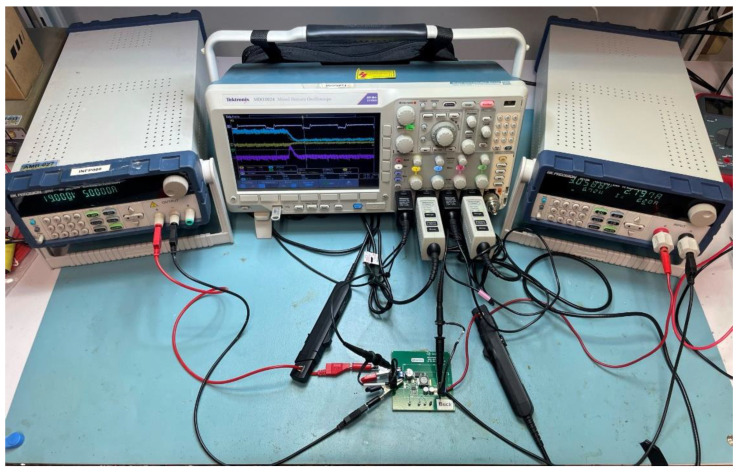
Experimental setup to acquire data from DC-DC converter.

According to the proposed method, the first step is to estimate the converter parameters before the aging process starts. To this end, the TRRNLS algorithm is applied using the signals acquired from the converter under steady state and when a load change occurs. Figure 10 presents the experimental setup used to acquire the data of the DC-DC converter.

Once the data have been measured and pre-processed, the parameters of the DC-DC converter are estimated using the optimization method presented in this paper. The parameter estimation algorithm was programmed in Matlab and Simulink. The initial, minimum, and maximum values were set based on the criteria detailed in the Section 2.3.1. Seven parameters for the steady state data and four parameters from the data acquired from the transitory were identified. The average error in the estimation of all parameters was 3.55%. The results from the estimations were *C* = 208.23 µF and ESR = 429.5 mΩ, while the values obtained using the LCR measuring device were *C* = 207.58 and ESR= 427.2 mΩ. This represents a relative error of 0.31% and 0.54%, respectively.

**Figure 11:**
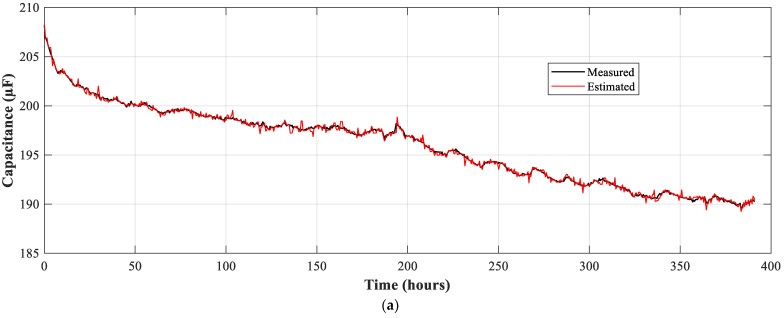

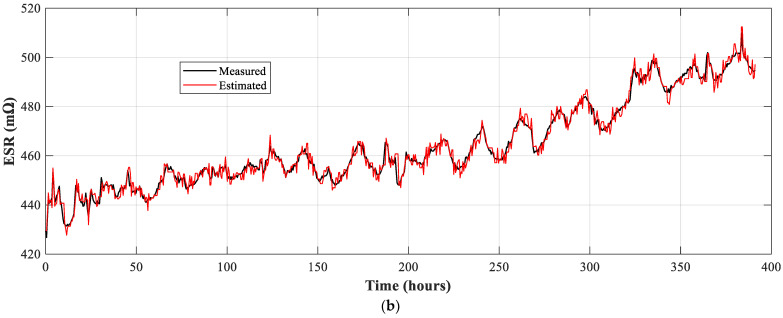
Measured and estimated values during the accelerated aging test. (**a**) Capacitance; (**b**) ESR.

Once the parameters have been estimated, the accelerated aging test begins. The test lasted for 391 h and measurements were performed every 30 min. It is important to mention that the electrolytic capacitor parameters were identified at each iteration based on the input and output signals. They were also measured using a LCR measuring device. Figure 11 presents the evolution of the capacitance and ESR during the degradation process. One curve refers to the actual value of these parameters, while the other curve shows the estimations done by means of the TRRNLS algorithm.

Figure 11 proves that the proposed optimization algorithm is capable of estimating the capacitor parameters with high accuracy during the accelerated aging test. The total number of estimated values was 753, which corresponds to the number of measurements performed during the aging process. The average estimation error is 0.081% for the capacitance and 0.367% for the ESR, which in both cases is considerably low.

The next step is to split the estimated data during the accelerated aging test into training, validation, and test sets in order to train the model proposed in Section 2.3. The training set considers the 70% of the original dataset, corresponding to 527 time steps or 275 h. The size of the validation set is a tenth part of the original time-series, which comprises 75 steps in the interval from 275 to 313.5 h. Finally, the test set is formed by the last 20% data of the capacitor, comprising the last 77.5 h of the aging test.

As mentioned in the previous section, once the data has been divided, the next step is to tune the set of hyper-parameters that enhances the accuracy of the model. To this end, a random search algorithm was applied, where 100 neural networks were trained for 10 epochs each. Table 1 presents the boundaries of the search algorithm and the tuned values.

Once the hyper-parameters have been tuned, the CNN-LSTM neural network is trained based on the strategy detailed in the previous section. The total number of epochs dealt with was 32, the model reaching convergence after 12.2 s.

**Figure 12:**
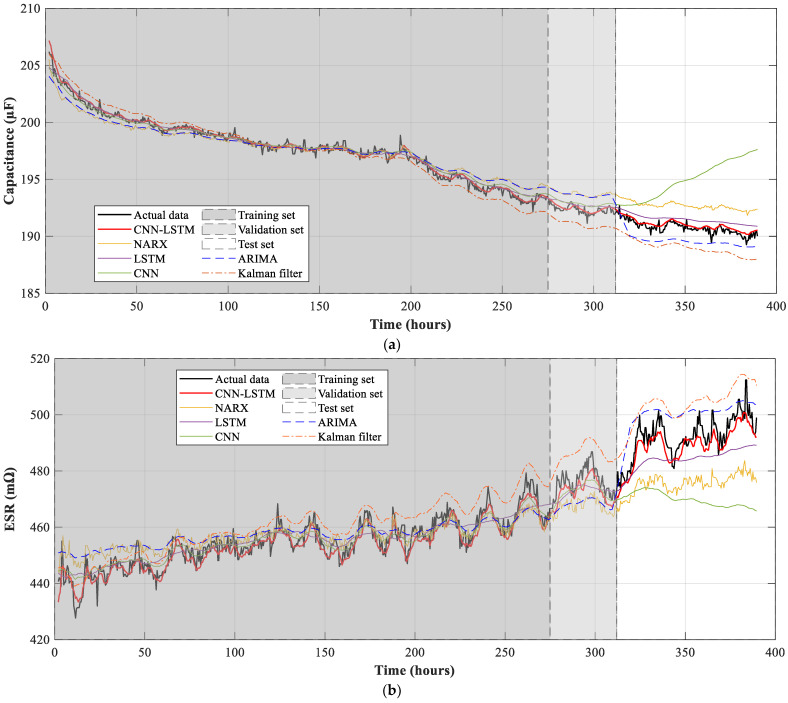
Prediction of the future values of (**a**) Capacitance; (**b**) Equivalent series resistance of the electrolytic capacitor.

Figure 12 and Table 2 summarize the results obtained after training the CNN-LSTM neural network and compare these results against those obtained by applying other approaches. The model accuracy is obtained using the test dataset, which includes data that have not been used in the training process. The performance indicators used to compare the results obtained are the RMSE and the coefficient of determination (*R*^2^) since both assess the quality of the regression when compared to the actual values [38]. All models were programmed and trained using Python and it was carried out by means of a GeForce RTX 2080 Super GPU.

From the results presented in Table 2, it is evident that the proposed approach is able of predicting the future outputs of the capacitance and ESR of the electrolytic capacitor. Results attained with the CNN-LSTM approach present a coefficient of determination that is almost 1 for the two predicted variables. On the other hand, the CNN-LSTM neural network outperforms the other approaches, since it presents a lower value of the RMSE and a value of the determination coefficient *R*^2^ closer to 1 in the predictions made using the test dataset. The higher accuracy of the proposed method is also appreciated in Figure 12, where the model is capable of replicating the behavior of the electrolytic capacitor, even when it is tested with a set of data that was not used in the training process. The training time of the proposed method is longer than that of the LSTM approach, but lower than that of the CNN neural network. However, the time elapsed is relatively low considering that the proposed model consists of two hidden layers with different architecture.

Once the model is trained, it is used to calculate the end of life (EOL) of the capacitor. This value is obtained by estimating the capacitance until its value reaches the 80% of its initial value (80% of 208.23 µF = 166.4 µF) or when the ESR reaches the 280% of the initial value (280% of 429.5 mΩ = 1202.6 mΩ). The EOL of the electrolytic capacitor calculated by the CNN-LSTM model is 1563 h due to the reduction of the capacitance to the 80% of its initial value. On the other hand, the ESR requires about 9212 h to reach its EOL value of 1202.6 mΩ.

**Figure 13:**
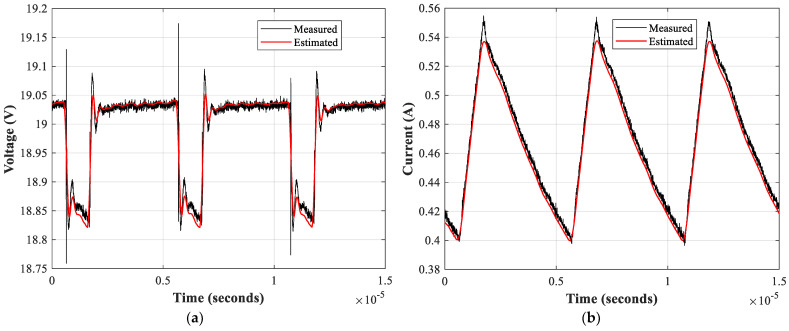

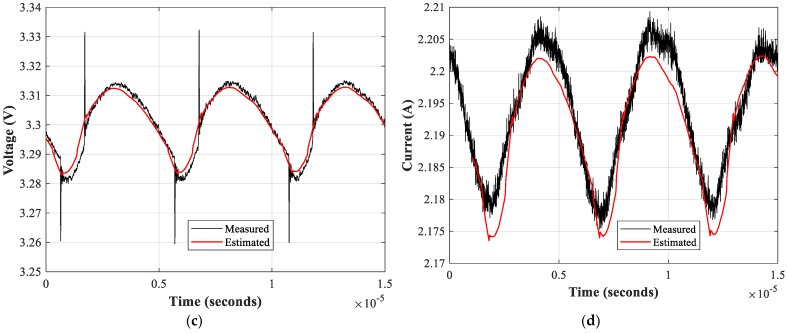
Measured signals versus the estimated ones with the CNN-LSTM NN. (**a**) Input voltage; (**b**) Input current; (**c**) Output voltage; (**d**) Output voltage.

Finally, a comparison between the measured and estimated voltages and currents of the DC-DC converter is performed with a 1.5 Ohm resistive load. To this end, experimental data are acquired from the laboratory setup once the accelerated aging test has finished. The estimation of these waveforms is done by taking the predicted values of the capacitance and ESR by means of the CNN-LSTM NN at the last time step from simulations of the converter considering the predicted values of parameters *C* and ESR. Figure 13 presents the four waveforms, where it is evident that the model replicates accurately the response of the converter.

## 4. Discussion

This study has proposed a method for identifying the state-of-health of the DC-DC converters that feed the electric machines that propel electric and hybrid electric vehicles. The method performs an offline and non-invasive estimation of the converter parameters by using the signals acquired at the input and output terminals of the converter. An accelerated aging test has been applied to degrade the electrolytic capacitor that is placed at the output of the converter. During this test, the parameters of the capacitor are identified by applying the aforementioned method. Finally, after the test finishes, a deep learning model is trained in order to predict the future health condition of the electrolytic capacitor, and thus, the DC-DC converter state-of-health.

The results presented in Section 3 show that the TRRNLS optimization algorithm identifies with high accuracy the electrolytic capacitor parameters during the aging test. The average error of the 751 estimations was lower than 1% for the two parameters identified in the process. This is very advantageous, because some manufacturers do not allow to measure the internal components of the power converters, while the proposed method is based on non-invasive measurements. Moreover, the process of gathering the data can be automated as it was explained in Section 2.

Once the value of the parameters was estimated, the data were used to train a CNN-LSTM neural network. The results show that after tuning the hyper-parameters, the trained model replicates with accuracy the behavior of the DC-DC converter when it suffers electrical overstress. The proposed approach outperforms other neural network topologies when the models are tested using a new set of data. The calculated performance indicators present a coefficient of determination of 0.99, which is desirable in a prediction problem. The waveforms presented in Figure 13 are the evidence that the combination of the parameter estimation algorithm and the prediction model lead to an accurate representation of the future input and output signals of the DC-DC converter.

In conclusion, the method proposed in this paper can estimate the actual and future values of the capacitance and ESR of the electrolytic capacitors used in DC-DC converters. The considered aging test was based on applying an electrical overstress to the electrolytic capacitor. It is noted that the same approach can be extended to other factors that may degrade this component, such as temperature, pressure, etc. This prognostics approach results helpful in the prevention of failures in the powertrain of the electric vehicle given the negative impact of a faulty electrolytic capacitor in a power system.

## Figures and Tables

**Table 1:** Random search algorithm to set the parameters of the CNN-LSTM NN.

Parameter	Minimum	Maximum	Optimal
Kernel size	1	15	4
LSTM neurons	1	100	31
*n*	20	100	56
*m*	1	40	12

**Table 2:** CNN-LSTM performance and comparison with other methods.

Method	Capacitance		ESR		Time Elapsed
	RMSE	*R* ^2^	RMSE	*R* ^2^	
CNN-LSTM	0.00042	0.995	0.0016	0.990	12.2 s
NARX	0.00080	0.825	0.0055	0.846	66 s
LSTM	0.00056	0.961	0.0035	0.944	8.03 s
CNN	0.00121	0.774	0.0094	0.790	12.8 s
ARIMA	0.00065	0.949	0.0032	0.951	4.7 s
Kalman filter	0.00071	0.938	0.0041	0.932	2.8 s
